# Free Tissue Transfer in Head and Neck Reconstruction: A Multidisciplinary 15-Year Experience

**DOI:** 10.3390/clinpract13040074

**Published:** 2023-07-12

**Authors:** Athanasios Papanikolaou, Laetitia Guarino, Roland Giger, Benoît Schaller, Mihai Constantinescu, Radu Olariu, Ioana Lese

**Affiliations:** 1 Department of Plastic and Hand Surgery, Inselspital, Bern University Hospital, University of Bern, 3010 Bern, Switzerlandradu.olariu@insel.ch (R.O.);; 2 Department of Otorhinolaryngology, Head and Neck Surgery, Inselspital, Bern University Hospital, University of Bern, 3008 Bern, Switzerland; 3 Department of Cranio-Maxillofacial Surgery, Inselspital, Bern University Hospital, University of Bern, 3010 Bern, Switzerland; 4 Department for BioMedical Research, University of Bern, 3010 Bern, Switzerland

**Keywords:** free flaps, outcomes, head and neck reconstruction

## Abstract

Background: Free tissue transfer is considered the gold standard in reconstruction of extensive defects in head and neck surgery. The aim of this 15-year retrospective study is to analyze the outcomes of free tissue transfers in the head and neck area in a tertiary referral university hospital. Materials and methods: A retrospective, single-center study of all patients undergoing free tissue transfers for head and neck reconstruction between 2006 and 2020 was performed. Patient demographics, comorbidities, flap characteristics, outcomes and complications were assessed. Results: A total of 353 free flaps were performed. The most common defect etiology was synchronous oncologic resection (74.2%). The majority of patients had at least one comorbidity (70.3%), with smoking recorded in 46.2% of the cases and alcohol consumption in 31.7%. The anterolateral thigh flap was the most commonly used flap (37.7%), followed by the osteoseptocutaneous fibula flap (26.9%). Our overall flap success rate was 97.7%, while the overall complication rate was 45.9%. Conclusions: Free tissue transfer in head and neck reconstruction is reliable. However, complication rates remain high due to the complexity of such cases and frequent presence of comorbidities. Nonetheless, when effectively managed within a multidisciplinary team, complications rarely jeopardize the overall reconstruction outcome.

## 1. Introduction

Reconstruction of complex head and neck defects by means of free tissue transfer is the gold standard in defect coverage after performing extensive oncologic resections [1,2]. Refinements in microsurgical techniques provide restoration of both form and function while decreasing the considerable morbidity once associated with large ablative defects. Moreover, free flaps are also preferred to reconstruct defects in secondary elective procedures—e.g., for the treatment of major osteoradionecrosis or unsatisfactory outcomes after local flap reconstructions, as well as in cases of extensive traumas that involve multiple structures in the head and neck region. The reasons for such a preference lie in the complexity of defects that is commonly encountered. Specifically, reconstruction plans may aim to address multiple goals at the same time: the need for soft tissue coverage of underlying structures such as bone, major vessels and nerves as well as the need for separation of them from non-sterile areas such as the oral and nasal cavity by reconstructing anatomical barriers [2], obliteration of dead space with well-vascularized tissue, reconstruction of bone defects while maintaining an acceptable functional result in terms of airway clearance and unhindered breathing, as well as oral competence and oral food intake. Last but not least, a high regard for an adequate aesthetic result should be kept, as such reconstructions concern a rather visible portion of the human body and may entail implications in the patient’s judgement of self-worth and body integrity as well as psychosocial behavior and perception of quality of life.

When striving for high-quality, safe and refined reconstructions in order to achieve the above-mentioned goals, it becomes obvious that conventional reconstructive approaches by means of local pedicled flaps represent a second line of reconstruction for major tissue defects after oncologic surgery. Such flaps may not be always able to provide tissue of sufficient quantity and/or quality and their harvesting may also be impaired due to previous surgery or radiotherapy in their donor region. Regional flaps, such as the pedicled latissimus dorsi flap, should not be fully disregarded, as they can still be valuable options when patients would not qualify for free tissue transfer, especially when adequate recipient vessels are not available [2].

Various workhorse flaps with presumably ideal properties accommodating defect requirements of tissue volume, texture and pedicle length have been proposed in the literature. The reliability of commonly used flaps can be predictable for an experienced reconstructive surgeon. However, the heterogeneous patient-related characteristics in the head and neck region can entail confounding factors that may interfere with postoperative complications and affect outcomes. Overall surgical complication rates in these patients still remain rather high, with reported total rates of up to 54% in the literature [3,4]. A better understanding of the association between various patient-related factors and postoperative complications is thus necessary in order to be able to better counsel the patients preoperatively and possibly refine reconstructive techniques. Therefore, the aim of this study is to analyze the reconstruction outcomes after free tissue transfers in the head and neck region over a 15-year period in a tertiary referral university hospital.

## 2. Materials and Methods

This retrospective study included all patients from 2006 to 2020 who underwent free tissue transfer for reconstruction of defects in the head and neck region treated in Inselspital, University Hospital Berne, Switzerland. Patients who underwent reconstruction of defects in the head and neck region with other types of flaps (such as pedicled flaps or random flaps) and other reconstruction modalities were not considered for this study. Each free flap was considered separately, even though in some cases some patients received more than one free flap. The following patient-related data were collected: patient age at the time of surgery, gender, comorbidities such as regular tobacco or alcohol consumption, body mass index and presence of obesity, American Society of Anesthesiologists (ASA) score [5], diabetes, high blood pressure, heart failure/coronary disease, cerebrovascular disease, coagulation disorders, peripheral arterial occlusive disease and chronic obstructive pulmonary disease. Furthermore, we assessed surgery-specific variables, such as etiology of defects, which entailed reconstruction at the time of tumor resection, elective reconstructions and trauma cases. The first of these groups consisted of those patients with various malignancies undergoing free tissue transfer immediately or at a maximum of 3 months after tumor resection. As elective reconstructions, we defined free tissue transfers for defects that were reconstructed more than three months after the initial tumor resection, chronic osteomyelitis with plate infection or other posttraumatic complications ensuing more than three months after the initial surgical intervention and osteoradionecrosis. The category of trauma cases consisted of those patients with soft-tissue trauma and fractures, including infections, undergoing reconstructive surgery immediately or at a maximum of 3 months after the incident. Other variables included anatomic location of defect, involvement of bone in the defect, flap used for reconstruction, vascular compromise of flaps and flap failure, and postoperative minor and major complications, including those of the donor and recipient site. Complications were classified as major when their management required a surgical intervention. The minimum follow-up period was 1 year.

Statistical analyses were performed using Prism 8.0 (GraphPad Software, San Diego, CA, USA). Student’s *t*-test or the Mann–Whitney test, depending on the distribution of the data, were used for continuous variables, while the Pearson’s X^2^ and Fischer’s test were employed for the categorical variables. The study was conducted in accordance with the Declaration of Helsinki, and approved by the Ethics Committee of Inselspital, Bern University Hospital.

## 3. Results

**Figure 1:**
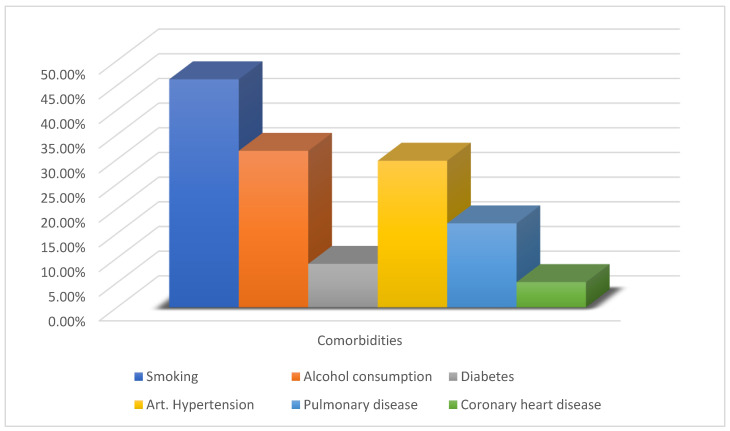
Patients’ most common comorbidities.

We assessed a total of 353 flaps in 346 patients that underwent reconstruction with free tissue transfer in the head and neck region from 2006 to 2020 in our unit. Among all cases, 65.2% of flaps were performed in male patients and 34.8% in females, with a mean age of 57.5 years (range: 6–89). Patients’ characteristics are outlined in Figure 1 and Table 1.

The defect etiology in the vast majority of cases (262 cases, 74.2%) was cancer resection with immediate reconstruction needed. Only 3 cases were reconstructed after trauma (0.8%), while the rest 88 flaps were considered elective indications (24.9%), such as delayed reconstructions (more than 3 months after tumor resections), dynamic reanimation for facial palsy and elective reconstructions of scalp defects. The anterolateral thigh flap (ALT) was utilized in 133 cases and was the most commonly used flap overall (37.7%), followed by the osteoseptocutaneous fibula flap in 93 cases (26.3%). A more detailed description of the various types of flaps used is presented in Table 2.

In regard to defect characteristics, the median bone defect size in patients that required such reconstruction was 9.4 cm (range: 4–25). Among all patients, 36.3% (128 cases) presented with a bone defect. However, only 75% of those defects (96 cases) required reconstruction with a bone flap, with 90 cases involving the mandible or/and the maxilla.

The majority of the flaps were performed in patients that had at least one comorbidity (70.3%), with the most common one being arterial hypertension (29.7%). Smoking was recorded in 46.2% of the cases, and alcohol consumption in 31.7% of them.

### 3.1. Vascular Complications of Flaps

Vascular compromise of a flap was observed in 5.4% of all cases (19 flaps) and venous thrombosis was responsible for the majority of these incidences (57.9%). However, 11 out of the 19 flaps were successfully salvaged through emergency surgical revision and as such, the overall flap success rate was 97.7%. We recorded therefore eight cases of flap failures which concerned seven patients in total. Of these patients, six were treated with a new salvage free flap (five ALT flaps and one latissimus dorsi flap), whereas in one patient, both the initial flap (scapula flap) and the salvage flap (fibula flap) failed and this patient`s maxilla defect was obliterated with a prosthesis in the end.

### 3.2. Overall Complications

When looking at the overall complications, we observed an incidence of 45.9%, corresponding to 162 cases. Complications were classified as major when a surgical intervention was required to treat them. In particular, this was deemed necessary for 105 cases (29.7%). While we encountered a low rate of major complications involving the donor site (6.2%), the incidence of major complications at the recipient site was approximately four times higher (24.9%). Complications at the donor site were mostly minor ones and as such they were treated conservatively, while complications at the recipient site were mostly major ones and therefore required surgical intervention. A more detailed description is outlined in Table 3. As anticipated, we observed an association of complications with the patients’ ASA score. More specifically, the overall complication rate increased with the ASA score (*p* < 0.001): ASA 1—24.5%, ASA 2—39.7%, ASA 3—72.1% and ASA 4—62.5% (However, there were only 16 cases with an ASA score of 4). Moreover, overall postoperative complications were significantly more common among smokers, as 51.5% of smokers sustained a complication compared with 41.1% of non-smokers (*p* = 0.049). However, age was not associated with an increased rate of complications. 

We observed a statistically significant association for complications in patients that underwent reconstruction with a bone flap (55.8%), compared to those patients that underwent reconstruction with another type of flap (42.2%, *p* = 0.024).

Between the categories entailing cases of tumor resection with immediate reconstruction (reconstruction in <3 months), trauma cases (reconstruction in <3 months) and elective cases (delayed reconstruction >3 months), we have not found any statistically significant differences regarding complications.

### 3.3. Donor Site Complications

In regard to the donor site, 6.2% of flaps (22 cases) showed a major complication, while 8.8% of flaps (31 cases) showed a minor one. The most common major complication at the donor site was tissue necrosis (10 cases), followed by major infection (5 cases) and major bleeding/hematoma and major wound dehiscence (4 cases each). While postoperative seromas at the donor site were also encountered, most of them were treated conservatively. Major and minor complication rates of the flap donor site are outlined in Table 4. Analyzing complication rates at the donor site between various flap types, we found that the rates for major and minor complications were significantly higher in patients that underwent reconstruction with bone flaps: 11.5% of these cases sustained a major complication (*p* = 0.045) and 14.6% a minor one (*p* = 0.017). More specifically, we observed that rates of infection and tissue necrosis of the donor site were significantly higher among bone flaps (5.2% (*p* = 0.023) and 12.5% (*p* < 0.001), respectively). However, postoperative seroma rates were significantly higher in the donor sites of muscle and musculocutaneous flaps (10.4% (*p* = 0.003)). Alcohol consumption in patients’ history was found to be significantly associated with donor site infection (4.5% vs. 0.8% in patients abstaining from alcohol, *p* = 0.044) as well as donor site tissue necrosis (8.1% vs. 2.9% in patients abstaining from alcohol, *p* = 0.019). Obesity as defined by an increased BMI > 30 kg/m^2^ was found to be significantly associated with wound dehiscence (7.4% vs. 2.4% in non-obese patients, *p* = 0.049) as well as infection at the donor site (7.4% vs. 1.5% in non-obese patients, *p* = 0.022).

### 3.4. Recipient Site Complications

As expected, the majority of complications, both major and minor ones, concerned the recipient site. Specifically, 24.9% of cases showed a major complication, while 15.3% of them showed a minor one at the recipient site. 

Looking at overall postoperative complications at the recipient site, major and minor wound dehiscence was the most frequent one (12.1% or 43 cases). However, upon examining complications that required surgical intervention, the most common major complication at the recipient site was partial flap necrosis (9.3% or 33 flaps), followed by major bleeding/hematoma (6.2% or 22 cases).

Major dehiscence (dehiscence needing surgical intervention in order to treat) was the third most common major complication at the recipient site with a rate of 5.9% (21 cases), demonstrating that despite wound dehiscence being the most common complication at the recipient site, almost half of these complications were managed conservatively.

Major infections were less common and were observed in only 5.4% of cases. Major and minor complication rates of the flap recipient site are outlined in Table 5. Upon analyzing complications of the recipient site, we did not detect any statistically significant differences between complication rates among cutaneous/fasciocutaneous flaps, muscle-/musculocutaneous flaps and bone flaps.

We also examined statistical associations between comorbidities and complication rates. In particular, the presence of a comorbidity was associated with increased incidence of both minor complications (19.8% rate vs. 4.8% rate in patients with no comorbidities, *p* < 0.001) and major complications (29.4% rate vs. 14.3% in patients with no comorbidities, *p* = 0.003) at the recipient site. Among all comorbidities, those most likely to potentially jeopardize the reconstruction result at the recipient site were the presence of diabetes and smoking. Diabetes was associated with significantly increased risk for vascular compromise of the flap (16.1% risk vs. 4.3% in patients without diabetes, *p* = 0.006) while smoking was significantly associated with partial flap necrosis at the recipient site (14.7% risk vs. 8.9% in non-smokers, *p* = 0.045).

## 4. Discussion

Our data demonstrate that free tissue transfer is a reliable reconstructive method for defects in the head and neck area with high success rates, despite frequent complications.

An overall flap success rate of 97.7% in our patient population is consistent with reported success rates of 94.8–98% in free tissue transfer by other authors [6,7,8]. Nevertheless, reconstructive surgeons involved in the treatment of such patients should be aware of the increased probability for postoperative complications, especially at the recipient site. While the most common complication at the recipient site in our patients was wound dehiscence, reconstructive surgeons may anticipate higher risks for partial flap necrosis in smokers as well as a higher risk for vascular compromise in diabetic patients.

Even when a major vascular compromise of a free flap may occur, immediate emergency flap revision might successfully salvage the flap and save the entire reconstruction. The rate of vascular compromise we found in our patients (5.4%) is comparable to the rates of vascular compromise described in the literature of 6.2% up to 9.9% [2,6,9].

Moreover, our findings in regard to salvage rates of 57.9% are also consistent with reported salvage rates of 63.7% as well as the fact that the majority of vascular compromise cases (58%) involved venous problems [2,6].

Extensive ablative defects in the head and neck area may typically result after oncologic resections for various malignancies. Meticulous patient selection is seldom possible in this patient population with substantial head and neck defects requiring reconstruction. The chance of at least one comorbidity being present is high among this patient demographic. These characteristics indicate an overall reduced health status; however, these specific patients require complex and extensive surgical reconstructions involving soft tissue and/or bone defects. Due to the entailed complexity, it has been advocated that such treatments may be performed in reference centers that can provide the expertise necessary for achieving optimal outcomes [10].

The majority of such procedures are required after oncologic resections and almost half of the patient population in need of them are smokers. Therefore, it is of clinical relevance for the multidisciplinary team treating such patients to consider comorbidities that may be associated with increased complication rates. This should be given due importance among many other valuable benefits of multidisciplinary preoperative assessment and treatment [11]. Moreover, it is well documented by many authors that a high ASA score correlates with increased postoperative complication rates in free tissue transfer [9,12,13,14,15] and our findings support this as well. Further risk factors that are associated with increased complication rates in free tissue reconstructions include age and smoking [12,16]. High overall complication rates are not uncommon in patients undergoing reconstruction in the head and neck area. We found overall complication rates of 45.9% in our patient cohort with a rate of 29.7% for major complications. Our findings are consistent with results published in the literature, with rates of 53% for overall complications and 40% for major complications [10].

Many studies have examined the role of diabetes mellitus in the incidence of complications in head and neck reconstructions using free tissue transfer [17,18,19]. Specifically in regard to vascular compromise, the incidence of diabetes mellitus was found to be significantly higher in cases of flap failure compared with those patients with successful free tissue transfer [20]. In accordance with such observations, our results demonstrate that diabetes mellitus was indeed associated with significantly higher incidence for vascular compromise (16.1%) compared with those patients without diabetes (4.3%, *p* = 0.006). Interestingly, such findings have led some authors to adapt their reconstructive approach by preferring pedicled flaps when addressing defect reconstructions in the head and neck area of diabetic patients [19]. However, there are also authors reporting no association between free flap failure and the presence of diabetes [21].

In regard to recipient site complications, Nao et al. found a 32% overall rate with the most frequent ones being infection, fistula and hematoma [22]. Our similar findings underline that recipient site complications in these patients are higher than the ones found in other localizations, mostly due to the special environment of the oral cavity. In patients undergoing free tissue transfer for defects of the lower extremity, reported complication rates range from 13.4% to 21.8% [23,24].

The fact that complication rates in free tissue transfer for head and neck defects is reported higher may be attributed to the nature of reconstruction of head and neck defects, commonly involving sterile and non-sterile anatomical areas. Free flap surgery in the head and neck area is commonly utilized after ablative procedures adjacent to the oral cavity and/or the upper respiratory tract. In such non-sterile areas, it is imperative for flap surgery to reconstruct anatomical barriers that are airtight and watertight while the flap and its sutured wound margins are constantly put under stress such as saliva, respiratory air pressure and mucosal secretions since the very beginning, from the flap inset. At a later timepoint, further stress to the reconstruction result might occur through oral fluid and food intake. Such factors can put constant strain on the flap and its margins to autochthonous tissues and invoke wound dehiscence or infections spreading from non-sterile to otherwise sterile tissue planes. These factors may in fact be unavoidable in reconstructions in the head and neck area and explain differences in complication rates compared to free tissue transfers on the lower extremity. Even though free flaps transferred on the lower extremity have to sustain other types of stress factors, this is generally not the case from the very beginning of the flap-healing phase. Immediate weight bearing, extremity movement and shearing forces are generally avoided during the early postoperative course by means of extremity fixation, meticulous wound dressings and careful ambulation. These measures aim to provide sufficient time for the flap and surgical wounds to heal adequately by protecting the newly transferred flap from possible stress that could jeopardize its function.

As far as donor site morbidity is concerned, we found an overall rate of 15% comprised of both minor and major complications, consistent with other authors reporting overall complication rates of 17% at the donor site [25], even though rates as high as 43.2% have been reported [15].

Since the first mandibular reconstruction with a free fibula flap by Hidalgo in 1989 [26], it has been shown that this flap is a reliable workhorse flap to be considered as a standard of care for head and neck defects requiring bone reconstruction [27].

Our findings demonstrate that free fibula flap is indeed the go-to method in the vast majority of cases requiring bone reconstruction in the head and neck area. Nevertheless, we observed that rates of infection and tissue necrosis of the donor site were significantly higher among bone flaps (5.2%, *p* = 0.023 and 12.5%, *p* < 0.001, respectively). Conversely, we found no significant difference in complication rates of the recipient site between bone flaps and other types of free flaps.

We acknowledge that this retrospective study examining outcomes in patients undergoing free tissue transfer for head and neck reconstructions has limitations. Pre- and postoperative radio- and chemotherapy has not been assessed, since an accurate and complete collection of the data over the 15-year period was not possible. The extended time period included in the study might also account for variations in treatment practices and indications, as well as involvement of different surgeons performing the mentioned reconstructions over the years. Furthermore, different flap preferences among surgeons may influence the indication for each case as well as the reconstructive outcomes.

Generalizing the outcomes to other types of flaps that were not commonly used in our unit can potentially be employed in head and neck reconstruction cases, but it needs to be completed with caution. Even though each treatment center tends to employ established workhorse flaps, surgeon-dependent preferences in flap selection and technique are unlikely to be homogenous among treatment centers. As such, this being a single-center study, a selection bias may be entailed.

Furthermore, we studied patients who underwent a variety of primary surgeries, so increased variability to outcomes is to be expected. Additionally, the seven cases undergoing salvage procedures with free flaps were treated as separate cases. Finally, the retrospective assessment and chart review may entail that subclinical or asymptomatic complications may have not been exhaustively recorded.

## 5. Conclusions

Free tissue transfer in the head and neck region is a very reliable method for defect reconstruction, even in complex cases. Complication rates might seem high in this patient population; however, this can be attributed to the unavoidable fact that such patients tend to have multiple comorbidities that prove to be considerable risk factors. It becomes evident that a proper multidisciplinary management of these patients is highly valuable and should be endorsed. When complications are anticipated and managed timely and effectively, they rarely jeopardize the reconstructive outcome. Therefore, microsurgical free tissue transfer remains the preferred method for optimal reconstruction of substantial defects in the head and neck area.

## Figures and Tables

**Table 1:** Patients’ characteristics.

Patients’ Characteristics		Number of Flaps (% of Total 353)
Gender	Male	230 (65.2%)
	Female	123 (34.8%)
Comorbidities	Presence of at least one comorbidity	248 (70.3%)
	History of smoking	163 (46.2%)
	History of alcohol consumption	112 (31.7%)
	Diabetes mellitus	31 (8.8%)
	Arterial hypertension	105 (29.7%)
	Heart failure	16 (4.5%)
	Coronary heart disease	18 (5.1%)
	Coagulation disorders	3 (0.8%)
	Pulmonary disease	60 (17%)
	Peripheral arterial occlusive disease	17 (4.8%)
ASA Score ^1^	1	49 (13.9%)
	2	131 (37.1%)
	3	157 (44.5%)
	4	16 (4.5%)
**Patients’ characteristics**	**Median (Range; IQR ^2^)**	
Age [years]	60 (6–89; 18)	
BMI ^3^ [kg/m^2^]	23.4 (14.49–36.73; 5.91)	
Bone defect size [cm]	9.4 (4–25; 5.8)	
Time until signs of bone consolidation postoperatively [weeks]	30 (3–185; 16.3)	

Abbreviations: ^1^ ASA Score—American Society of Anesthesiologists Score, ^2^ IQR—interquartile range, ^3^ BMI—Body Mass Index.

**Table 2:** Flap types.

Flap Characteristics		Number of Flaps (%)	
Flap type	Fasciocutaneous and cutaneous	ALT ^1^ 133/353 (37.7%)	209/353 (59.2%)
		SCIP ^2^ 26/353 (7.4%)	
		Scapular/parascapular 26/353 (7.4%)	
		Radial forearm 16/353 (4.5%)	
		Other 8/353 (2.2%)	
	Muscle and myocutaneous	Latissimus dorsi 38/353 (10.8%)	48/353 (13.6%)
		Gracilis 10/353 (2.8%)	
	Osseous and osteocutaneous	Fibula 93/353 (26.3%)	96/353 (27.2%)
		Other osteocutaneous flap (radial forearm, scapular/parascapular) 3/353 (0.8%)	

Abbreviations: ^1^. ALT—anterolateral thigh flap, ^2^. SCIP flap—superficial circumflex iliac artery perforator flap.

**Table 3:** Incidence of Complications.

		Number of Flaps (% of Total 353)
Overall	Overall complications	162 (45.9%)
	Major complications	105 (29.7%)
	Minor complications	74 (21%)
Donor site	Major complications	22 (6.2%)
	Minor complications	31 (8.8%)
Recipient site	Major complications	88 (24.9%)
	Minor complications	54 (15.3%)

**Table 4:** Types of complications at the donor site.

		Number of Flaps (% of Total 353)
Donor site	Minor infection	2 (0.6%)
	Major infection	5 (1.4%)
	Minor tissue necrosis	6 (1.7%)
	Major tissue necrosis	10 (2.8%)
	Minor bleeding/hematoma	9 (2.5%)
	Major bleeding/hematoma	4 (1.1%)
	Minor seroma	9 (2.5%)
	Major seroma	2 (0.6%)
	Minor wound dehiscence	6 (1.7%)
	Major wound dehiscence	4 (1.1%)
	Other major complications	1 (0.3%)
	Other minor complications	5 (1.4%)

**Table 5:** Types of complications at the recipient site.

		Number of Flaps (% of Total 353)
Recipient site	Minor infection	9 (2.5%)
	Major infection	19 (5.4%)
	Minor partial flap necrosis	8 (2.3%)
	Major partial flap necrosis	33 (9.3%)
	Minor bleeding/hematoma	13 (3.7%)
	Major bleeding/hematoma	22 (6.2%)
	Minor seroma	4 (1.1%)
	Major seroma	0 (0%)
	Minor wound dehiscence	22 (6.2%)
	Major wound dehiscence	21 (5.9%)
	Other minor complication	7 (2%)
	Other major complication	0 (0%)

## Data Availability

The data presented in this study are available upon request from the corresponding author.
